# A two-stage deep learning-based hybrid model for daily wind speed forecasting

**DOI:** 10.1016/j.heliyon.2024.e41026

**Published:** 2024-12-12

**Authors:** Shahab S. Band, Rasoul Ameri, Sultan Noman Qasem, Saeid Mehdizadeh, Brij B. Gupta, Hao-Ting Pai, Danyal Shahmirzadi, Ely Salwana, Amir Mosavi

**Affiliations:** a Future Technology Research Center, National Yunlin University of Science and Technology, Douliu, Taiwan; b Department of Information Management, International Graduate School of Artificial Intelligence, National Yunlin University of Science and Technology, Douliu, Taiwan; c Computer Science Department, College of Computer and Information Sciences, Imam Mohammad Ibn Saud Islamic University (IMSIU), Riyadh, 11432, Saudi Arabia; d Computer Science Department, Faculty of Applied Science, Taiz University, Taiz, 6803, Yemen; e Water Engineering Department, Urmia University, Urmia, Iran; f Department of Computer Science and Information Engineering, Asia University, Taichung, 413, Taiwan; g Symbiosis Centre for Information Technology (SCIT), Symbiosis International University, Pune, India; h Center for Interdisciplinary Research, University of Petroleum and Energy Studies (UPES), Dehradun, India; i Department of Big Data Business Analytics, National Pingtung University, Pingtung, Taiwan; j Graduate School of Engineering Science and Technology, National Yunlin University of Science and Technology, 123 University Road, Douliou, 64002, Yunlin, Taiwan; k Institute of Visual Informatics, Universiti Kebangsaan Malaysia, 43600, Bangi, Selangor, Malaysia; l John von Neumann Faculty of Informatics, Obuda University, Budapest, Hungary; m Ludovika University of Public Service, Budapest, Hungary

**Keywords:** Wind speed, Forecasting, Gradient boosting, Long short-term memory, Machine learning, Artificial intelligence, Big data, Data science

## Abstract

Global adoption of wind energy continues to increase, while improving the efficiency of turbine settings requires reliable wind speed (WS) models. The latest models rely on artificial intelligence (AI) optimizations which constructs tests on a range of novel hybrid models to examine the reliability. Gradient Boosting (GB), Random Forest (RF), and Long Short-Term Memory (LSTM) are used in new combinations for data pre-processing. A Time Varying Filter-based Empirical Mode Decomposition (TVFEMD) model is coupled with the GB and LSTM standalone models, to create TVFEMD-GB and TVFEMD-LSTM hybrids, which are run in competition with each other. Eventually, a preferred hybrid form is established, simultaneous hybridization of TVFEMD with GB and LSTM. This study is the first to hybridize these fundamental systems, and create a TVFEMD-GB-LSTM model that can forecast WS. This study finds that the novel hybrid models exhibit superior performance to standalone GB and LSTM models, opening the pathway to alternative WS prediction techniques.

## 1 Introduction

Modelling the wind power is crucial to improve the efficiency of this renewable energy source as discussed in several studies, e.g., Refs. [[1], [2], [3], [4], [5]]. As the energy generated using wind is considered clean and renewable, it has gained widespread popularity [[6], [7], [8]]. Increasing electricity consumption is leading to more demand for efficiency [9,10]. Thus, researchers are examining new ways to improve renewable energy resources. Wind power is a promising and regenerative resource for energy production [[11], [12], [13], [14]]. Wind speed (WS) is a key meteorological parameter, and an important variable in climatology, oceanography, and other renewable energy-related fields. In particular, effective and efficient wind power generation is closely connected to accurate WS measurement [15,16], and precise WS forecasting assists with fault detection in wind turbines [17].

Given the importance and relevance of WS, researchers now focus to develop techniques to accurately forecast WS. Accurate WS forecasts can be challenging to anticipate, due to the sporadic, or random characteristics of wind [3,[18], [19], [20], [21]]. Predictive approaches utilize various time scales and methods, for example forecasts are required for both short (*i.e.* hourly and daily) and long-term (*i.e.* monthly and yearly) time scales [22,23]. Short-term wind speed prediction is of great importance, to reduce scheduling errors in wind energy systems; which in turn affects grid reliability and market-based ancillary services [[22], [23], [24]]. Long-term WS forecasts are valuable when selecting new sites for wind turbines, and influence engineering decisions such as optimal blade angle and weight [22,23,25]. Thus, short and long-term wind speed predictions are of vital importance, and have unique applications [22,23].

WS forecasting approaches are divided into physical models [26], traditional statistical methods [27], and artificial intelligence (AI) or machine learning (ML) techniques [28]. Physical models require precise mathematical illustrations, depend on weather data, and can be used for long-term modeling over large geographical regions. The traditional approaches are now giving way to statistical learning models that involve multidimensional and nonlinear equations [29]. Rapid development of AI frameworks, and related techniques such as ML and deep learning, have already been used for WS forecasting [30,31].

Xiao et al. [32] recommend a self-adaptive kernel extreme-learning machine (ELM), and reports accurate performance for WS prediction. Yan et al. [33] predict WS via a long short-term memory (LSTM) model and its hybrids, with seasonal autoregressive integrated moving average (SARIMA) and ensemble empirical mode decomposition (EEMD). The authors report that the hybrid methods accurately forecast WS. Yang et al. [34] test the potential of four decomposition methods, coupled with the ELM, to predict WS. They highlight the superiority of the EEMD approach. Duan et al. [35] propose a hybrid paradigm, combining EMD convolutional neural networks (CNNs), and recurrent neural networks (RNNs), concluding that the hybrid approach exhibits a superior forecasting ability. Zhang et al. [36] compare the classic Gaussian process regression (GPR) and variational heteroscedastic GPR (VHGPR). They note that the VHGPR improves the point WS forecasts, and yields superior WS predictions to GPR.

Chen et al. [37] couple LSTM with the EEMD and a genetic algorithm (GA) to predict WS. Their findings highlight the superiority of their proposed model, over the other standalone and coupled models. Liu et al. [38] propose a coupled deep-learning-based RNN paradigm for WS prediction, and the results exhibit satisfactory performance. Li et al. [39] use a novel hybrid framework based on wavelets, an Elman neural network (ENN), and boosting algorithms to predict WS. The coupled models outperformed the standalone frameworks. Additionally, Li et al. [40] use an innovative boosted approach known as fuzzy time series, that is optimized via a multi-objective algorithm, to balance model stability and accuracy.

Marndi et al. [41] compare the LSTM framework with SVM and ELM, and note that LSTM predicts WS with greater accuracy. To enhance forecasting performance, Zhang et al. [42] utilize an adaptive boosting (AdaBoost) technique, based on variational mode decomposition (VMD), fruit fly optimization (FFA), ARIMA, and a deep belief network. Their adaptive model was shown to be superior to other methods. Hu et al. [43] propose a coupled model integrating VMD with differential evolution (DE) and an echo state network (ESN) (*i.e.* VMD-DE-ESN) for forecasting WS. Their hybrid framework demonstrated satisfactory performance. Dosdoğru and İpek [44] develop a hybrid approach for predicting hourly WS based on extreme gradient boosting, AdaBoost, and ANN, calibrated using particle swarm optimization (PSO). The coupled method yielded practicable results. Ding et al. [45] propose a new WS forecasting tool based on double decomposition, piecewise error correction, ENN, and ARIMA. The hybrid technique exhibits enhanced prediction performance, compared to various standalone methods. Gan et al. [46] use temporal convolutional networks (TCNs) to realize interval estimation for WS forecasting. The TCNs exhibit enhanced predictive ability and reliability compared to classic ANNs and canonical RNNs.

Hua et al. [47] propose a VMD based approach involving partial least squares, improved atom search optimization, and ELM. The proposed model outperforms the benchmark models for short-term WS prediction. Emeksiz and Tan [48] use a hybrid model consisting of EEMD adaptive noise (CEEMDAN), local mean decomposition, the Hurst model, and a backpropagation neural network (BPNN). The proposed model outperforms conventional forecasting methods. Wang et al. [49] combine ELM with the AdaBoost algorithm for WS forecasting with data from neighboring sites. The method yields more accurate results than standalone models. Parri and Teeparthi [50] suggest a new framework that integrates successive VMD, transformer, and query selection procedures to predict short-term WS. The model exhibits robust performance relative to other comparable approaches. A coupled model is proposed by Bommidi et al. [51], which predicts WS by combining a transformer model with an improved complete ensemble empirical mode decomposition with adaptive noise (ICEEMDAN). The model outcomes indicate that the hybrid model has superior performance. Álvarez-Rodríguez et al. [52] propose a novel concept-bottleneck model, and prove its viability during periods of extreme WS variation. A hybrid model proposed by Wu et al. [53] merges two decomposition techniques (ICEEMDAN; empirical wavelet transform (EWT)) with temporal fusion transformers and adaptive differential evolution; however, this is also augmented by the option of an external (data) archive. This study outcome exhibits the highest accuracy of the developed models for interpretable WS forecasting. Peng et al. [54] implement a coupled method integrating the VMD and attention gated recurrent unit; the study reports suitable performance of the proposed hybrid model. Qin et al. [55] implement a hybrid system that combines LSTM with Fuzzy Entropy and EEMD; their paper reports that the proposed hybrid framework exhibits superior performance.

Given the aforementioned literature results, standalone models typically appear inferior to well-tuned coupled or hybrid methods [56]. This indicates that metaheuristic algorithms and data pre-processing are of value when constructing a hybrid model. There are several key pre-processing schemes, and these are: wavelet theory, EMD, EEMD, VMD, etc. One pre-processing approach rarely seen in WS prediction literature involves Time Varying Filter-based Empirical Mode Decomposition (TVFEMD). TVFEMD represents an effective mathematical technique to decompose continuous-time signals or functions into components of various scales.

Given the models explored in the literature, and the continually growing need for accurate WS prediction, this study conducts the following investigation: (1) forecast daily WS time series for three sites located in IL, USA, *i.e.* Carbondale, Champaign, and DeKalb, using the individual Gradient Boosting (GB), Random Forest (RF), and Long Short-Term Memory (LSTM); (2) develop two hybrid models, by individually hybridizing the TVFEMD with the GB and LSTM (*i.e.* TVFEMD-GB and TVFEMD-LSTM); then (3) propose a two-stage hybrid scheme through simultaneous integration of TVFEMD, GB, and LSTM (*i.e.* TVFEMD-GB-LSTM), and finally; (4) compare the relative forecasting performance of the standalone and novel hybrid methods. Some error indices and schematic diagrams are employed to comprehensively evaluate model performance. There is existing research that couples different pre-processing frameworks with AI models to forecast WS – but the key novel approach of this study is the individual coupling of TVFEMD with GB and LSTM, then simultaneously hybridizing the TVFEMD, GB, and LSTM systems. Specifically, the proposed two-stage hybrid model (TVFEMD-GB-LSTM) obtains the benefit of both the TVFEMD and GB during the establishment of LSTM.

## 2 Methodology

### 2.1 Data and study areas

**Fig. 1:**
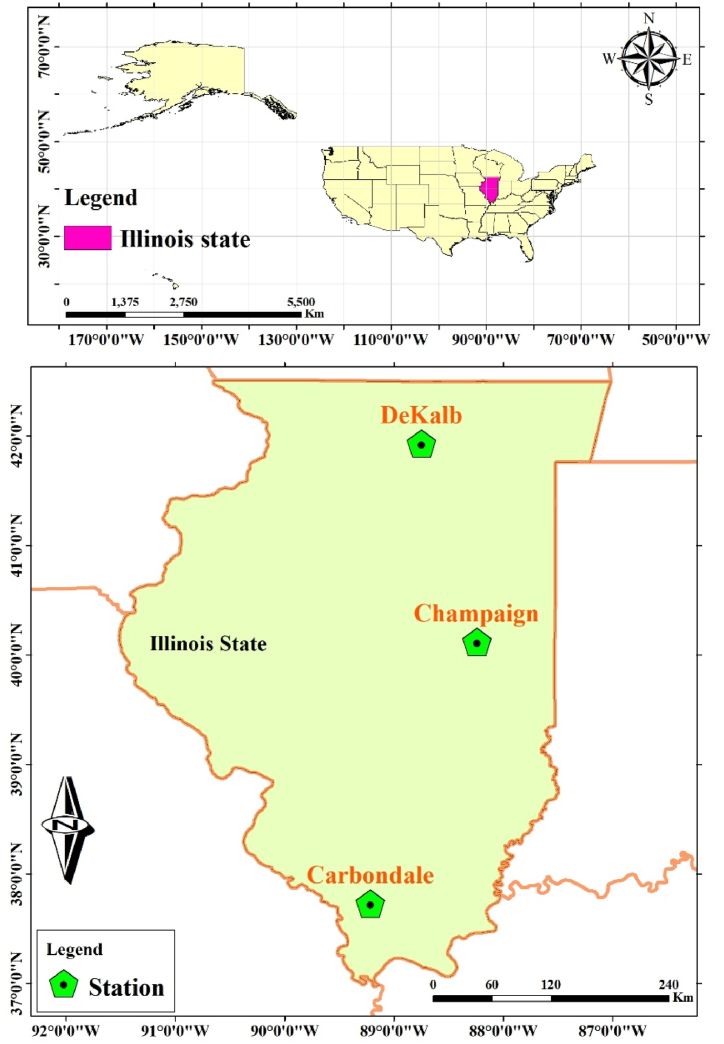
Geographical location of the three WS stations in IL, USA.

The three study areas, Carbondale (latitude: 37.73 °N, longitude: 89.22 °W, altitude: 137 m), Champaign (latitude: 40.12 °N, longitude: 88.24 °W, altitude: 219 m), and DeKalb (latitude: 41.93 °N, longitude: 88.75 °W, altitude: 265 m) are all located in IL, USA. Fig. 1 shows the geographical locations. According to the Köppen climate classification system, the respective climates of Carbondale, Champaign, and DeKalb, are humid subtropical, humid continental, and humid continental.

**Fig. 2:**
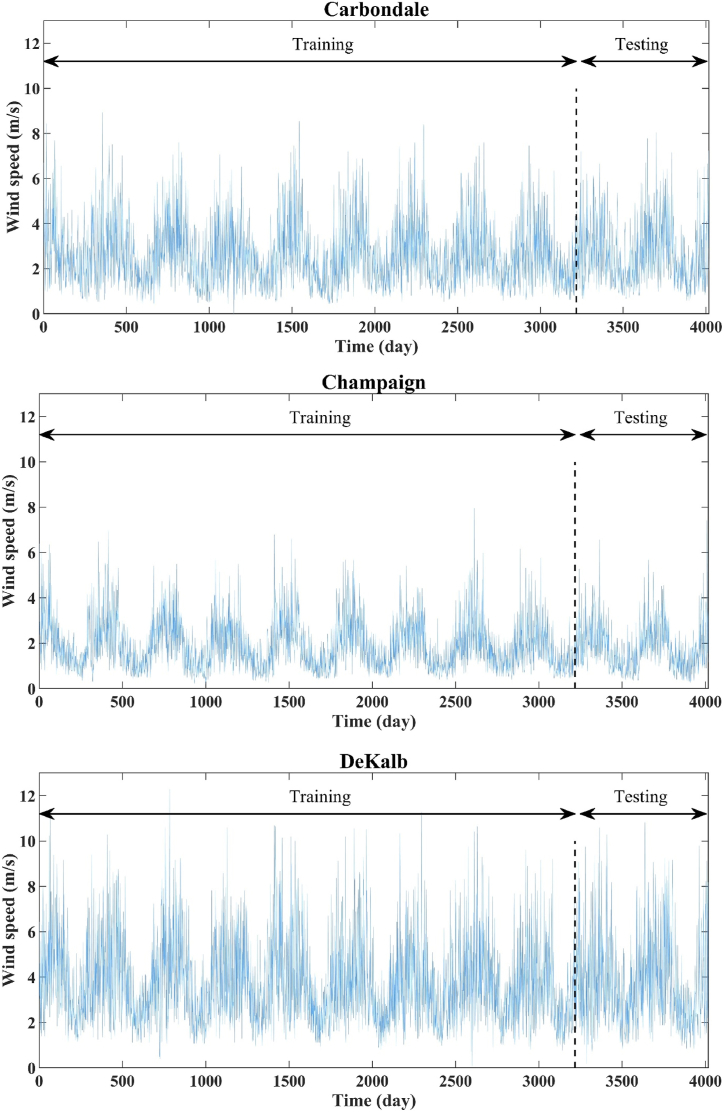
Time series of the daily wind speed data for the study sites.

WS observations from the three IL sites are daily time-series, and run from January 1, 2012 to December 31, 2022. The data can be obtained from https://www.isws.illinois.edu, and there are 4,018 observations. The first 3,217 observations (*∼*80 % of the whole dataset) are used for training, and the remaining 801 (∼20 %) are used for testing. The daily data for the three locations are plotted in Fig. 2. The statistical characteristics of the daily WS data are presented in Table 1. The statistical parameters during training and testing are almost the same. Taking into account the average and standard deviations present in Table 1, higher values are observed at DeKalb, and relatively lower values are observed at Champaign. As a whole, the WS data exhibits a positive skewness.

**Table 1:** Statistical properties of the daily wind speed data at the studied stations.

Station	Period	Minimum (m/s)	Maximum (m/s)	Average (m/s)	Standard deviation (m/s)	Coefficient of variation	Skewness
Carbondale	Training	0	8.941	2.598	1.365	0.525	1.025
	Testing	0.581	8.047	2.667	1.359	0.510	0.902
Champaign	Training	0.179	7.957	1.885	1.065	0.565	1.144
	Testing	0.268	7.421	1.828	1.009	0.552	1.152
DeKalb	Training	0.089	12.294	3.710	1.812	0.488	0.991
	Testing	0.179	10.818	3.747	1.938	0.517	0.950

### 2.2 Models

#### 2.2.1 Gradient boosting

Boosting operates sequentially, unlike bagging, and each subsequent prediction model is built using the results of the previous one, until the desired number of models is reached. The enhanced prediction result of each new model is then presented as the eventual single output. Gradient boosting, GB [57], is a prominent technique within ensemble learning, and transforms weak learners into strong ones by progressively refining predictions through a series of weaker trees. Unlike random forest, GB constructs weaker trees, with each subsequent tree improving upon the output of the previous one. These collectively reinforce each other, ultimately yielding a robust result.

#### 2.2.2 Random forest

Ensemble learning algorithms are also prominent in AI and prediction-based academic research. Ensemble learning involves creating multiple models and aggregating their results for a final output, rather than relying on a single forecasting model. Bagging and boosting approaches are the primary strategy in ensemble learning. Random Forest (RF) and Gradient Boosting (GB) are well-known examples of bagging and boosting approaches.

RF models were developed by Breiman [58], but have recently gained considerable popularity because of their robust performance, ease of implementation, and low computational cost. It is an ensemble learning technique that constructs multiple regression trees. Each tree is trained using a bootstrap sample extracted from the entire training set, where the sample is successively divided into branches through threshold tests, comparing randomly selected features to set thresholds. This iterative process continues until terminal or leaf nodes contain a single data point or a pre-set number of data points. Each leaf node aggregates its corresponding output training data points, typically through averaging, to yield the final value. Validation data points traverse the tree during the training stage, and if the output leads to a satisfactory score, the tree is validated. The training process stops when the forest reaches the user-specified size, with the ensemble of regression values contributing to the final regression value, often determined through averaging. More information about RF can be found in Ref. [58].

#### 2.2.3 Long short-term memory

Long short-term memory (LSTM) network is an elaboration of the recurrent neural network (RNN) method [59], which has already been extensively researched due to its excellent performance during time-series forecasting. RNNs consist of three layers: an input layer, a hidden layer, and an output layer. Neurons in the hidden layer possess a self-recurrent connection property. This property allows them to receive both the current input value and the output value, calculated by the hidden layer at the previous time step. However, traditional RNNs face some limitations, particularly in long-term dependent learning tasks, where gradient back-propagation can lead to gradient vanishing and explosion issues. LSTM networks effectively mitigate these traditional RNNs issues. An essential advancement in LSTM networks, compared to RNNs, is incorporating LSTM cells, each equipped with unique memory capabilities, which enhances the ability of the network to maintain information over extended periods.

The LSTM cells utilize three gates: the forget gate, the input gate, and the output gate. Each gate performs a specific function, enabling the LSTM network to regulate the transmission state, and remember information crucial for long-term memory tasks, while discarding irrelevant data. LSTM networks capture and retain important patterns in sequential data using these gates; thus, this approach is well-suited for applications requiring robust long-term memory capabilities.

#### 2.2.4 Time varying filter-based empirical mode decomposition

Empirical Mode Decomposition (EMD), proposed by Huang et al. [60], is a time-frequency analysis method that decomposes signals based on their inherent time scale information. Unlike Fourier transform and wavelet transform, which depend on selecting a basis function, EMD employs an adaptive decomposition approach to eliminate the need to choose a basis function. Therefore, EMD is suitable for both linear and nonlinear signals, as it can decompose the original data into intrinsic mode functions (IMFs) and residual components according to their respective time scales.

Traditional EMD is prone to mode aliasing. To overcome this, researchers [61] introduced the Time-Varying Filtering-based Empirical Mode Decomposition (TVFEMD) method, an improvement on conventional EMD. TVFEMD utilizes time-varying filters to address mode aliasing while preserving the sequence's time variability. This method performs time-varying filtering on the input sequence, by adapting the local cut-off frequency based on instantaneous amplitude and frequency. Finally, it decomposes the sequence into local high-frequency and low-frequency components to extract the IMFs. Recently, TVFEMD has been employed as in a variety of modelling applications. More details regarding the computation method of TVFEMD can be found in Ref. [61].

### 2.3 Model development

**Fig. 3:**
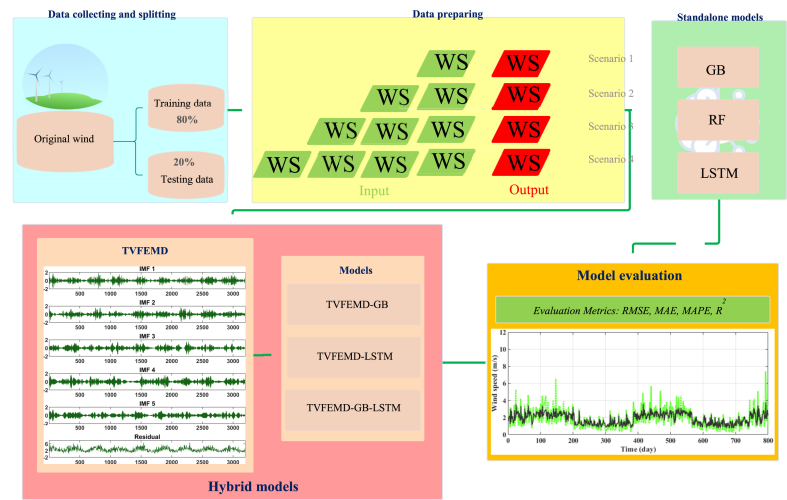
Process flow of this study.

The daily WS (WS_t_) time series of the study locations were forecast in this study. The models inputs were the lagged daily WSs, ranging from one to four lags (WS_t-1_, WS_t-2_, WS_t-3_, WS_t-4_), as indicated in Table 2. First, the standalone GB, RF, and LSTM frameworks were established to forecast the WS, and the relevant parameters were determined through trial-and-error. Next, three types of coupled methods were developed. A pre-processing TVFEMD technique was separately hybridized with the GB and LSTM for developing the TVFEMD-GB and TVFEMD-LSTM hybrid models. Finally, a two-stage TVFEMD-GB-LSTM hybrid model was developed by simultaneously merging the TVFEMD, GB, and LSTM. Fig. 3 shows the process flow of this research, indicating the different steps of WS forecasting by the proposed models. In the hybridized models, three different models, GB, LSTM, and GB-LSTM, were applied to forecast each IMF. In GB-LSTM, one IMF was considered to be forecasted by GB and the remainder by LSTM to reduce time while maintaining the accuracy of the WS forecasting. Finally, the sum of forecasted IMFs is considered as the final forecasted output. In this study, the grid search from the Neural Network Intelligence (NNI) [62] package was used to optimize the parameters of GB and LSTM. The main parameters of GB, such as the number of estimators and learning rate, and the parameters of LSTM, including the number of neurons, learning rate, batch size, and epochs, were optimized using the grid search algorithm. Additionally, the selected IMF for forecasting by GB was chosen through the grid search approach. For example, in the case of the Champaign station and the fourth scenario, the number of estimators and learning rate for GB were set to 100 and 0.0271 respectively, while the parameters for LSTM included 128 neurons, a learning rate of 0.0003, a batch size of 32, and 500 epochs. Furthermore, in the TVFEMD-GB-LSTM model, the sixth IMF was chosen for forecasting by GB.

**Table 2:** Input configurations defined in this study.

Model notation	Models	Inputs	Output
M1	GB1, RF1, LSTM1, TVFEMD-GB1, TVFEMD-LSTM1, TVFEMD-GB1-LSTM1	WS_t-1_	WS_t_
M2	GB2, RF2, LSTM2, TVFEMD-GB2, TVFEMD-LSTM2, TVFEMD-GB2-LSTM2	WS_t-1_, WS_t-2_	WS_t_
M3	GB3, RF3, LSTM3, TVFEMD-GB3, TVFEMD-LSTM3, TVFEMD-GB3-LSTM3	WS_t-1_, WS_t-2_, WS_t-3_	WS_t_
M4	GB4, RF4, LSTM4, TVFEMD-GB4, TVFEMD-LSTM4, TVFEMD-GB4-LSTM4	WS_t-1_, WS_t-2_, WS_t-3_, WS_t-4_	WS_t_

To optimize the parameters of the TVFEMD-GB-LSTM model for better performance, one should focus first on the preprocessing stage. The TVFEMD technique should accurately decompose the time series into IMFs, with the selection of IMFs guided by metrics like variance contribution or energy entropy. Fine-tune the decomposition parameters to capture relevant frequency components. For the hybrid model, assign low-frequency IMFs to GB and high-frequency IMFs to LSTM based on their respective strengths in handling smooth trends versus temporal dependencies. Grid search optimization can systematically determine the best IMF distribution, as well as key parameters like learning rate and the number of estimators for GB.

For the LSTM component, tune the number of neurons, batch size, learning rate, and epochs to balance complexity and training efficiency is a key point. Use early stopping to avoid overfitting and cross-validation to assess model robustness. The combination of GB and LSTM should minimize forecasting errors for each IMF, with the final forecast derived from their summation. Validation through metrics such as RMSE or MAPE ensures alignment with performance goals. This iterative process, supported by tools like Neural Network Intelligence (NNI), provides a comprehensive framework for achieving robust and accurate predictions.

### 2.4 Performance assessment metrics

The forecasting performance of the standalone and coupled models was evaluated using four error indices including root mean square error (RMSE), mean absolute error (MAE), mean absolute percentage error (MAPE), and determination coefficient (R^2^) as statistical error metrics, which can be mathematically expressed as follows:

(1) $$RMSE=\sqrt{\frac{\sum_{i=1}^{N}{\left(WS_{o,i}-WS_{f,i}\right)}^{2}}{N}}$$

(2) $$MAE=\frac{\sum_{i=1}^{N}\left|WS_{o,i}-WS_{f,i}\right|}{N}$$

(3) $$MAPE=\left(\frac{1}{N}\sum_{i=1}^{N}\left|\frac{WS_{o,i}-WS_{f,i}}{WS_{o,i}}\right|\right)\times 100\%$$

(4) $$R^{2}={\left(\frac{\sum_{i=1}^{N}\left(WS_{o,i}-\bar{WS_{o}}\right)\cdot \left(WS_{f,i}-\bar{WS_{f}}\right)}{\sqrt{\sum_{i=1}^{N}{\left(WS_{o,i}-\bar{WS_{o}}\right)}^{2}\cdot \sum_{i=1}^{N}{\left(WS_{f,i}-\bar{WS_{f}}\right)}^{2}}}\right)}^{2}$$

where $WS_{o,i}$ and $WS_{f,i}$ indicate the *i*th observed and forecasted daily WSs, respectively; $\bar{WS_{o}}$ and $\bar{WS_{f}}$ denote the mean values for the observed and forecasted daily WSs, respectively; and *N* illustrates the total number of observational values. A superior model corresponds to lower RMSE, MAE, and MAPE, and a higher R^2^.

## 3 Results

The forecasting performance of the individual and hybrid models are evaluated using four error metrics, RMSE, MAE, MAPE, and R^2^. Subsequently, the best-performing hybrid models are visualized using scatter, time series, Taylor, and violin diagrams. All simulations are performed using Python 3.8 and the tensorflow package, on the same machine, with a 3.61 GHz processor and 64 GB memory.

The daily WS time series data are forecast using the single and hybrid models. The input predictors are the antecedent daily WS data from one to four lags, as displayed in Table 2. Table 3, Table 4, Table 5 display the error metrics of the single GB, RF, and LSTM models, as well as the hybrid TVFEMD-GB, TVFEMD-LSTM, and TVFEMD-GB-LSTM models. At all three locations, the standalone models forecast the daily WS using lagged daily WS data. The RMSE, MAE, MAPE, and R^2^ metrics at Carbondale, in the testing phase, range from 1.162 m/s (GB2 and GB3) to 1.289 m/s (RF2), from 0.897 m/s (GB3) to 0.994 m/s (RF2), from 41.093 % (LSTM4) to 46.752 % (RF2), and from 0.269 (GB2) to 0.181 (RF2), respectively. The corresponding values for the Champaign station vary between 0.795 m/s (LSTM4) to 0.948 m/s (RF2), between 0.592 m/s (LSTM4) to 0.710 m/s (RF2), between 42.285 % (LSTM4) and 48.381 % (RF3), and between 0.380 (LSTM4) and 0.220 (RF2). Finally, the DeKalb station data varies from 1.729 m/s (LSTM4) to 1.896 m/s (RF2), from 1.358 m/s (LSTM3) to 1.470 m/s (RF2), from 48.459 % (LSTM3) to 52.729 % (RF2), and from 0.203 (LSTM4) to 0.112 (RF2). This demonstrates that the individual models do not exhibit acceptable performance when forecasting WS data, due to the relatively high RMSE, MAE, and MAPE values, and the low R^2^ values. Furthermore, all the standalone models yield similar error metrics during the test stage.

**Table 3:** RMSE, MAE, R^2^, and MAPE error metrics for the individual and hybrid models at Carbondale.

Model type	Model	Training				Testing			
		RMSE (m/s)	MAE (m/s)	MAPE (%)	R^2^	RMSE (m/s)	MAE (m/s)	MAPE (%)	R^2^
Individual	GB1	1.151	0.888	44.676	0.286	1.167	0.906	42.193	0.262
	GB2	1.121	0.863	43.508	0.324	1.162	0.898	41.594	0.269
	GB3	1.105	0.850	42.722	0.345	1.162	0.897	41.453	0.268
	GB4	1.079	0.830	41.741	0.377	1.164	0.901	41.764	0.267
	RF1	1.135	0.878	44.039	0.305	1.191	0.920	42.848	0.232
	RF2	0.612	0.458	22.790	0.821	1.289	0.994	46.752	0.181
	RF3	0.460	0.354	17.522	0.924	1.227	0.950	43.862	0.211
	RF4	0.447	0.342	16.982	0.932	1.194	0.924	42.771	0.237
	LSTM1	1.180	0.909	45.601	0.249	1.167	0.908	42.147	0.262
	LSTM2	1.157	0.890	44.608	0.279	1.165	0.904	41.743	0.265
	LSTM3	1.114	0.864	43.966	0.333	1.165	0.900	42.420	0.264
	LSTM4	1.077	0.819	39.853	0.379	1.205	0.912	41.093	0.224
Hybrid	TVFEMD-GB1	0.813	0.619	29.336	0.644	0.884	0.681	30.311	0.576
	TVFEMD-GB2	0.309	0.242	11.992	0.950	0.352	0.278	12.895	0.934
	TVFEMD-GB3	0.334	0.261	12.871	0.941	0.382	0.303	13.853	0.921
	TVFEMD-GB4	0.282	0.222	11.049	0.958	0.345	0.270	12.529	0.936
	TVFEMD-LSTM1	0.850	0.644	30.293	0.610	0.870	0.669	29.713	0.589
	TVFEMD-LSTM2	0.331	0.269	14.123	0.948	0.344	0.275	13.598	0.941
	TVFEMD-LSTM3	0.216	0.164	7.806	0.975	0.233	0.178	8.076	0.971
	TVFEMD-LSTM4	0.089	0.068	3.239	0.996	0.101	0.077	3.553	0.995
	TVFEMD-GB1-LSTM1	0.849	0.643	30.238	0.612	0.872	0.670	29.716	0.588
	TVFEMD-GB2-LSTM2	0.330	0.267	14.014	0.947	0.345	0.276	13.640	0.940
	TVFEMD-GB3-LSTM3	0.222	0.168	7.933	0.974	0.248	0.189	8.499	0.967
	TVFEMD-GB4-LSTM4	0.126	0.095	4.389	0.992	0.142	0.111	4.969	0.989

**Table 4:** RMSE, MAE, R^2^, and MAPE error metrics for the individual and hybrid models at Champaign.

Model type	Model	Training				Testing			
		RMSE (m/s)	MAE (m/s)	MAPE (%)	R^2^	RMSE (m/s)	MAE (m/s)	MAPE (%)	R^2^
Individual	GB1	0.835	0.626	42.393	0.381	0.849	0.641	45.266	0.295
	GB2	0.834	0.630	42.936	0.387	0.818	0.622	45.227	0.343
	GB3	0.787	0.593	39.836	0.453	0.814	0.610	43.707	0.350
	GB4	0.813	0.613	41.720	0.420	0.808	0.609	44.325	0.360
	RF1	0.836	0.628	42.471	0.379	0.844	0.638	45.039	0.302
	RF2	0.502	0.368	25.427	0.787	0.948	0.710	48.342	0.220
	RF3	0.341	0.256	17.010	0.922	0.886	0.682	48.381	0.269
	RF4	0.327	0.245	16.367	0.931	0.857	0.652	45.836	0.306
	LSTM1	0.861	0.648	43.618	0.342	0.820	0.621	44.331	0.339
	LSTM2	0.848	0.639	42.961	0.362	0.816	0.613	44.061	0.347
	LSTM3	0.837	0.627	42.011	0.378	0.804	0.601	43.042	0.366
	LSTM4	0.826	0.620	41.699	0.395	0.795	0.592	42.285	0.380
Hybrid	TVFEMD-GB1	0.400	0.309	20.794	0.860	0.669	0.518	35.471	0.570
	TVFEMD-GB2	0.288	0.219	15.131	0.936	0.304	0.224	16.388	0.921
	TVFEMD-GB3	0.217	0.168	11.402	0.959	0.256	0.192	13.164	0.936
	TVFEMD-GB4	0.305	0.233	16.122	0.927	0.322	0.243	17.743	0.909
	TVFEMD-LSTM1	0.605	0.443	28.109	0.675	0.585	0.432	29.536	0.663
	TVFEMD-LSTM2	0.217	0.164	10.829	0.959	0.207	0.160	10.961	0.958
	TVFEMD-LSTM3	0.143	0.108	7.056	0.982	0.138	0.105	7.364	0.981
	TVFEMD-LSTM4	0.057	0.043	2.922	0.997	0.056	0.044	3.154	0.997
	TVFEMD-GB1-LSTM1	0.597	0.437	27.892	0.684	0.590	0.438	30.067	0.657
	TVFEMD-GB2-LSTM2	0.217	0.164	10.827	0.958	0.209	0.163	11.159	0.957
	TVFEMD-GB3-LSTM3	0.139	0.105	6.879	0.983	0.140	0.105	7.392	0.981
	TVFEMD-GB4-LSTM4	0.106	0.078	4.962	0.990	0.097	0.076	5.089	0.991

**Table 5:** RMSE, MAE, R^2^, and MAPE error metrics for the individual and hybrid models at DeKalb.

Model type	Model	Training				Testing			
		RMSE (m/s)	MAE (m/s)	MAPE (%)	R^2^	RMSE (m/s)	MAE (m/s)	MAPE (%)	R^2^
Individual	GB1	1.601	1.247	42.814	0.220	1.758	1.381	49.215	0.175
	GB2	1.548	1.204	41.356	0.275	1.763	1.376	49.090	0.172
	GB3	1.541	1.196	40.998	0.281	1.754	1.370	48.684	0.181
	GB4	1.529	1.182	40.488	0.294	1.741	1.368	48.533	0.193
	RF1	1.577	1.224	41.852	0.243	1.790	1.402	49.428	0.153
	RF2	0.814	0.612	21.029	0.833	1.896	1.470	52.729	0.112
	RF3	0.657	0.505	16.981	0.920	1.833	1.420	50.669	0.134
	RF4	0.630	0.487	16.704	0.933	1.791	1.406	50.107	0.159
	LSTM1	1.639	1.276	43.815	0.182	1.744	1.369	48.889	0.188
	LSTM2	1.630	1.270	43.608	0.192	1.736	1.369	49.227	0.196
	LSTM3	1.597	1.236	42.191	0.224	1.741	1.358	48.459	0.192
	LSTM4	1.595	1.235	42.165	0.227	1.729	1.365	48.635	0.203
Hybrid	TVFEMD-GB1	1.094	0.836	26.987	0.636	1.335	1.015	34.707	0.525
	TVFEMD-GB2	0.396	0.312	10.189	0.952	0.527	0.410	13.569	0.926
	TVFEMD-GB3	0.458	0.360	11.748	0.937	0.590	0.463	15.445	0.908
	TVFEMD-GB4	0.420	0.329	10.829	0.948	0.551	0.429	14.213	0.920
	TVFEMD-LSTM1	1.156	0.878	28.372	0.593	1.313	0.998	33.908	0.539
	TVFEMD-LSTM2	0.449	0.348	11.156	0.939	0.500	0.386	12.852	0.933
	TVFEMD-LSTM3	0.311	0.238	7.598	0.971	0.365	0.285	9.681	0.965
	TVFEMD-LSTM4	0.135	0.102	3.330	0.995	0.160	0.121	4.164	0.993
	TVFEMD-GB1-LSTM1	1.154	0.876	28.306	0.595	1.314	0.998	33.949	0.539
	TVFEMD-GB2-LSTM2	0.444	0.345	11.060	0.940	0.511	0.396	13.181	0.930
	TVFEMD-GB3-LSTM3	0.332	0.255	8.219	0.967	0.387	0.303	10.539	0.960
	TVFEMD-GB4-LSTM4	0.169	0.129	4.236	0.991	0.218	0.162	5.548	0.987

Table 3, Table 4, Table 5 demonstrate that the forecasting performance of models M1–M4 at whole the studied locations did not exhibit significant differences. In other words, the introduction of additional inputs could not enhance the model performance but incurred additional runtime.

This study was also aimed at enhancing the forecasting accuracy of the daily WS data by developing three kinds of hybrid techniques. To this end, the pre-processing method of TVFEMD was separately coupled over the standalone GB and LSTM to obtain TVFEMD-GB and TVFEMD-LSTM hybrid frameworks. Moreover, a two-stage coupled scheme was established and proposed by simultaneously integrating the TVFEMD, GB, and LSTM to implement the TVFEMD-GB-LSTM hybrid paradigm.

The statistical indicators for all the three hybrid models developed for the Carbondale, Champaign, and DeKalb stations are represented in Table 3, Table 4, Table 5, respectively. The RMSE, MAE, MAPE, and R^2^ for the coupled models at Carbondale station (Table 3) in the test period ranged from 0.101 m/s (TVFEMD-LSTM4) to 0.884 m/s (TVFEMD-GB1), from 0.077 m/s (TVFEMD-LSTM4) to 0.681 m/s (TVFEMD-GB1), from 3.553 % (TVFEMD-LSTM4) to 30.311 % (TVFEMD-GB1), and from 0.995 (TVFEMD-LSTM4) to 0.576 (TVFEMD-GB1), respectively. Table 4, Table 5 also show that the ranges for the corresponding values are as RMSE: 0.056 m/s – 0.669 m/s, MAE: 0.044 m/s – 0.518 m/s, MAPE: 3.154 %–35.471 %, R^2^: 0.997–0.570 for the TVFEMD-LSTM4 and TVFEMD-GB1 models at Champaign station, as well as RMSE: 0.160 m/s – 1.335 m/s, MAE: 0.121 m/s – 1.015 m/s, MAPE: 4.164 %–34.707 %, R^2^: 0.993–0.525 for the TVFEMD-LSTM4 and TVFEMD-GB1 models at DeKalb station.

Performance comparisons of the single GB and RF with TVFEMD-based hybrid TVFEMD-GB and TVFEMD-LSTM models for the three study areas clearly demonstrated that the TVFEMD is a robust pre-processing tool to enhance the efficiency of GB and LSTM for forecasting the daily WS data. In the standalone models, all the data are input to the models without any pre-processing. In contrast, the TVFEMD decomposes the data into a series of newly decomposed datasets. Consequently, TVFEMD-based hybrid methods can increase the accuracy of standalone models. In general, the TVFEMD-LSTM models outperformed the TVFEMD-GB methods for all the stations because of improved error metrics.

In addition to the TVFEMD-based coupled models, another type of hybrid model was also established in this study. To achieve this, TVFEMD, GB, and LSTM were simultaneously coupled with each other to construct the TVFEMD-GB-LSTM hybrid paradigm. The performance evaluation of the individual GB and LSTM with proposed two-stage hybrid model at studied sites clearly indicated that the simultaneous coupling of the TVFEMD, GB, and LSTM helped to enhance the forecasting of the daily WS data than that of the WS forecasts of simple GB and LSTM. Additionally, taking into account the statistical metrics for the TVFEMD-GB-LSTM and TVFEMD-GB models listed in Table 3, Table 4, Table 5, the reliable capability of the proposed two-stage TVFEMD-GB-LSTM merged scheme to improve the forecasting performance of one-stage TVFEMD-GB hybrid method can be clearly confirmed.

Overall, the hybrid TVFEMD-LSTM models developed at the study locations presented better WS forecasts compared to the other types of standalone and coupled models. The models that achieved the most accurate WS forecasts in the testing phase for the Carbondale, Champaign, and DeKalb were TVFEMD-LSTM4 (RMSE = 0.101 m/s, MAE = 0.077 m/s, MAPE = 3.553 %, R^2^ = 0.995), TVFEMD-LSTM4 (RMSE = 0.056 m/s, MAE = 0.044 m/s, MAPE = 3.154 %, R^2^ = 0.997), and TVFEMD-LSTM4 (RMSE = 0.160 m/s, MAE = 0.121 m/s, MAPE = 4.164 %, R^2^ = 0.993), respectively. The improvement percentages of the RMSE, MAE, MAPE, and R error statistics for the mentioned superior hybrid models compared to their standalone models (*i.e.* LSTM4) were 91.62 %, 91.56 %, 91.35 %, and 77.49 % at Carbondale, respectively, 92.96 %, 92.57 %, 92.54 %, and 61.89 % at Champaign, respectively, as well as 90.75 %, 91.14 %, 91.44 %, and 79.56 % at DeKalb, respectively. These findings highlighted that the WSs forecasted using the proposed hybrid models exhibited enhanced accuracy.

As already concluded, the performance of individual models was not significantly improved via increasing the number of input predictors. In return, utilizing more delays as the models inputs could lead to better WS forecasts through the whole hybrid models than when fewer inputs were used.

**Fig. 4:**
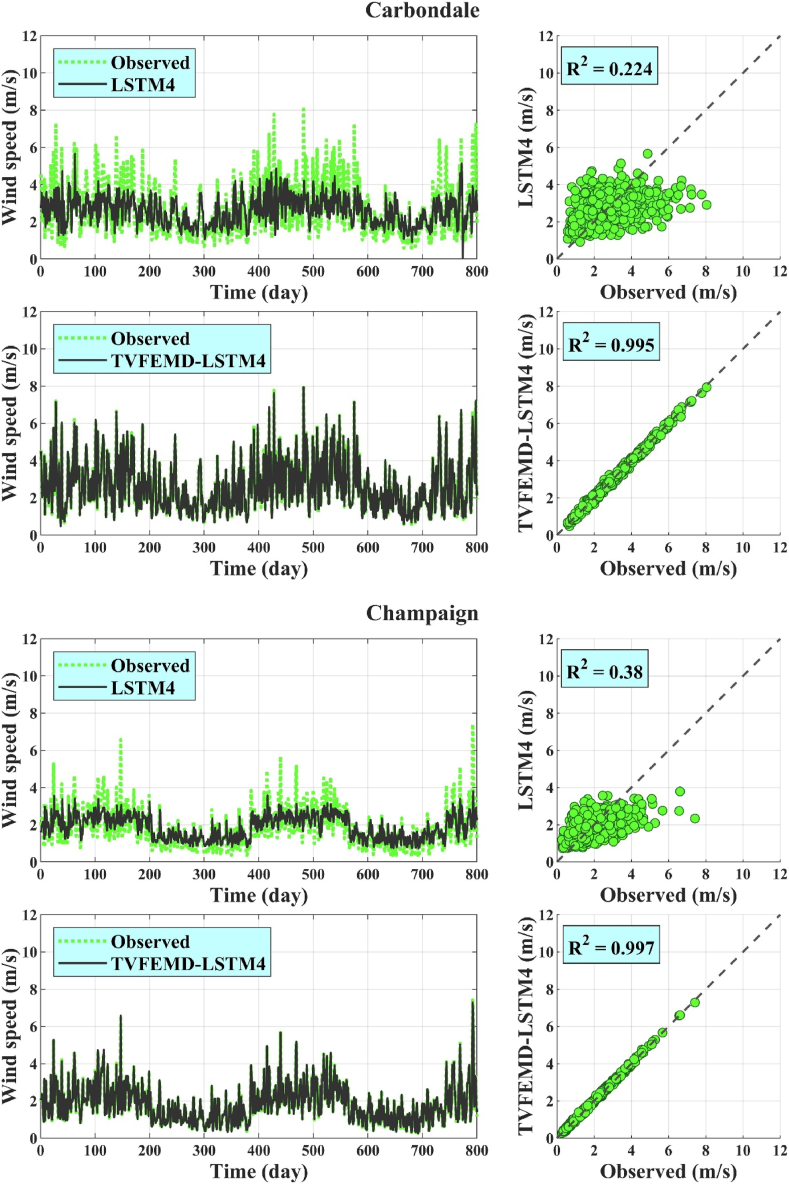

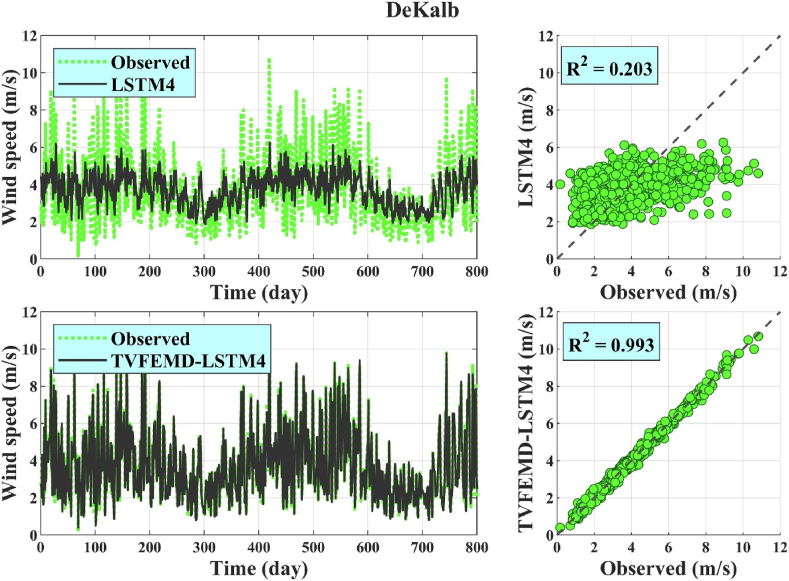
Scatter and time series plots of the observed and forecasted WS data obtained using the highest-performing hybrid models and corresponding standalone methods.

Finally, scatter, time series, Taylor, and violin plots were used to visually evaluate the model performances. In this analysis, the WS forecasts obtained using the best coupled models during the test phase (*i.e.* TVFEMD-LSTM4) and corresponding standalone methods (*i.e.* LSTM4) at each location were considered. The scatter plots (Fig. 4) demonstrated the superiority of the highest-performing hybrid models over the standalone models in forecasting the daily WS data. Specifically, the WS data for the best hybrid models exhibited less scattering around the dashed exact line. This finding is consistent with the higher R^2^ values for the hybrid models compared with those of the standalone models. The time series plots depicted in Fig. 4 also clearly show the higher performance of proposed TVFEMD-LSTM4 hybrid models compared to simple LSTM4 ones in forecasting observed WS data and following the trends of those data.

**Fig. 5:**
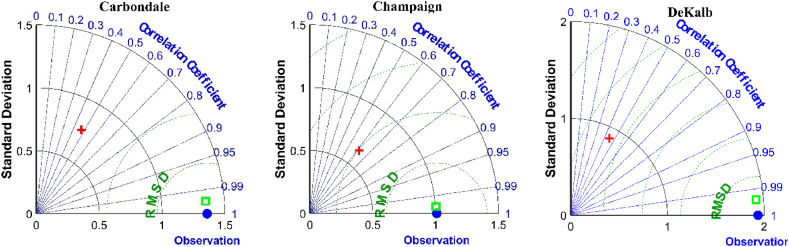
Taylor plots of the observed and forecasted WS data obtained using the highest-performing hybrid models, and corresponding standalone methods.

**Fig. 6:**
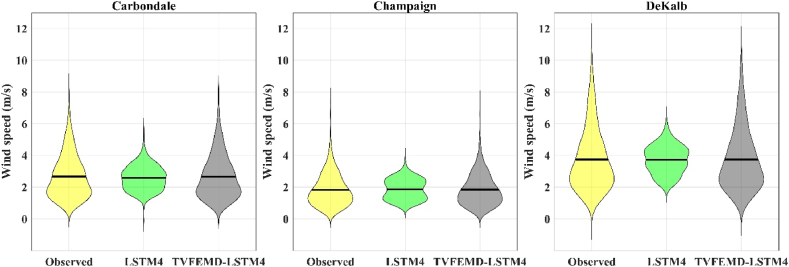
Violin plots of the observed and forecasted WS data, obtained using the highest-performing hybrid models and corresponding standalone methods.

Fig. 5 depicts the Taylor diagrams for the superior hybrid models and their relevant individual methods in the test stage for the studied locations. In this diagram, the root mean square difference (RMSD), correlation, and standard deviation of the forecasted values through the predictive models are compared with the actual values. The circular dashed green lines show the RMSD values, which the point corresponding to the observational reference data is shown in the center of its circles. The dashed blue lines that are the radii of the quarter circle of the Taylor diagram show the values of the correlation coefficient. The correlation coefficients related to the proposed hybrid models in the study stations are in the range of 1 to 0.99, while the correlations of the simple LSTM4 models are located between 0.4 and 0.7. A comparison of the standard deviation of the values predicted by the hybrid model with the observational data also denotes the dependable ability of the proposed hybrid model to estimate the standard deviation of the observed WS data than the simple model. In general, the closeness of the point of the hybrid model (*i.e.* green square) to the corresponding point of the observational data (*i.e.* blue circle) indicates the superior performance of the proposed TVFEMD-LSTM hybrid model in forecasting the WS data of the study stations. Meanwhile, the points related to the simple model are far away from the observational data, which represents the poor performance of these models. Furthermore, the violin plots (Fig. 6) demonstrates that the statistical distribution of the WS data for the highest-performing TVFEMD-LSTM4 coupled models was closer and similar to the observed data compared with those of the individual LSTM4 models. Additionally, the higher and lower bounds of the WS data could be better forecasted using hybrid models than standalone ones.

## 4 Discussion

This study proposes a two-stage hybrid method, merging TVFEMD and GB, with an LSTM, to target improved WS forecasting. The novel hybrid model exhibits better performance than the standalone GB and LSTM models, and this is explained by the fact that the TVFEMD-GB-LSTM hybrid model combines the strengths of TVFEMD preprocessing, using a robust machine learning technique of gradient boosting, and a deep learning method of LSTM. The TVFEMD decomposition effectively separates the raw wind speed data into intrinsic mode functions (IMFs) which lets capture both high-frequency and low-frequency components. By assigning low-frequency IMFs to GB which excels in handling smooth trends and high-frequency IMFs to LSTM which handles temporal dependencies, the hybrid model contains the strengths of both. This systematic hybridization improves forecasting accuracy and reduces errors compared to standalone models, which process the entire dataset without such decomposition or specialization. Additionally, the grid search optimization ensures that each model's parameters are finely tuned for better integration and overall performance. Coupled TVFEMD-based methods such as TVFEMD-GB and TVFEMD-LSTM are superior for WS prediction than standard GB and LSTM models. Both TVFEMD-GB and TVFEMD-LSTM outperform standalone GB and LSTM models due to the preprocessing step provided by TVFEMD. The TVFEMD decomposition isolates distinct patterns in the data, enabling each model to focus on specific data characteristics. For instance, GB, which performs better with smooth and low-frequency data, and LSTM, which is suited for high-frequency and sequential data, can work on the decomposed IMFs most aligned with their strengths. This targeted approach improves accuracy and reduces computational burden. Without TVFEMD, standalone models handle all data indiscriminately, limiting their ability to adapt to varying frequency scales and patterns, leading to higher errors and less precise forecasts.

The application of pre-processing (signal decomposition) techniques to improve WS forecasts has already been studied. For example, Moreno et al. [63] uses two decomposition methods, VMD and singular spectral analysis (SSA), to couple on a variety of machine/deep learning models. Their findings indicate that such hybrid methods outperform the standalone approaches. Two signal decomposition schemes, EMD and discrete wavelet transform (DWT), were coupled with three ML models, in a study by Katipoğlu [64]. The hybrid models exhibited superior forecasting ability than the simple models. Phan and Nguyen [65] utilize nested CEEMDAN to create a CNN-BiLSTM-based hybrid model; the model was dependable precision for WS prediction. Sibtain et al. [66] establish a two-stage hybrid method by hybridizing both the VMD and ICEEMDAN with an LSTM. They conclude that the hybrid model is superior for WS prediction, relative to the other models tested. Kumar and Yadav [67] test three types of signal decomposition: empirical WT (EWT), VMD, and Fourier, and these were then appended to an LSTM. The findings reveal that the developed hybrid models exhibit better accuracy than the standard WS. In Ref. [68] authors also focused on data decomposition, trend extraction, and predictive modeling, demonstrating the applicability of advanced time-series techniques across diverse climatological domains for improving decision-making in energy and hazard management.

As models grow in scope and ability, the hybridization process leads to time complexity issues. Accordingly, this study assesses the models developed and tested here; to accomplish this, the running times for the models are extracted and tabulated in Table 6. Considering the computational time of the individual models, LSTM requires more time than GB and RF, although in terms of performance they are all similar, and unsatisfactory. Among the developed hybrid models, the TVFEMD-GB run-time is shorter than the TVFEMD-LSTM and TVFEMD-GB-LSTM models; however, it also produces more errors than the other two coupled models, particularly TVFEMD-GB3 and TVFEMD-GB4. In terms of statistical accuracy, the TVFEMD-LSTM models are slightly better than the TVFEMD-GB-LSTM models; however, the run-time of the TVFEMD-GB-LSTM models are shorter than the TVFEMD-LSTM ones. This appears to be a key benefit of the two-stage hybrid TVFEMD-GB-LSTM model.

**Table 6:** Run-time (seconds) for the standalone and hybrid models.

Model	Station		
	Carbondale	Champaign	DeKalb
GB1	0.065	0.119	0.162
GB2	0.140	0.100	0.151
GB3	0.200	0.218	0.137
GB4	0.233	0.150	0.252
RF1	0.142	0.257	0.355
RF2	0.528	0.318	0.551
RF3	0.705	0.864	0.483
RF4	0.886	0.584	0.883
LSTM1	30.061	35.057	45.896
LSTM2	142.261	16.149	16.046
LSTM3	17.591	29.064	15.831
LSTM4	61.611	79.811	59.246
TVFEMD-GB1	14.545	15.088	17.395
TVFEMD-GB2	14.916	14.896	15.616
TVFEMD-GB3	15.019	16.049	15.450
TVFEMD-GB4	16.165	15.999	16.866
TVFEMD-LSTM1	170.391	346.290	274.234
TVFEMD-LSTM2	888.819	111.248	109.359
TVFEMD-LSTM3	108.377	186.337	109.529
TVFEMD-LSTM4	376.490	493.464	373.692
TVFEMD-GB1-LSTM1	125.224	240.866	212.049
TVFEMD-GB2-LSTM2	742.296	96.893	93.843
TVFEMD-GB3-LSTM3	90.359	157.988	94.833
TVFEMD-GB4-LSTM4	318.860	411.220	345.764

One limitation of this study, is that only the TVFEMD pre-processing method was used to generate the hybrid models. There remains significant scope to investigate hybrid models with different pre-processing techniques. For example, integrating different ML and deep learning techniques, with diverse optimization algorithms with data pre-processing may further improve efficiency and accuracy. Specifically, decomposition-based approaches, such as maximum overlap DWT, successive VMD, and diverse variants of EMD, appear to be promising approaches to WS prediction; and they are relatively easy to couple with machine/deep learning methods. The models in this study are only based on observational WS lags. Future studies may include other climate data as inputs, to improve forecasting outcomes.

## 5 Conclusions

Daily WS data fom Carbondale, Champaign, and DeKalb weather observation stations in Illinois, USA, were forecast using individual (GB, RF, and LSTM) and coupled hybrid models. Lagged WS data, from one to four lags, were fed into the models. The performance of the developed models was comprehensively evaluated by RMSE, MAE, MAPE, and R^2^. First, a TVFEMD pre-processing framework was separately hybridized with GB and LSTM in isolation. The hybrid TVFEMD-GB and TVFEMD-LSTM methods outperformed the individual GB and LSTM models. Subsequently, TVFEMD, GB, and LSTM were simultaneously integrated, to establish a TVFEMD-GB-LSTM scheme. Similar to TVFEMD-GB and TVFEMD-LSTM, the proposed two-stage hybrid method exhibited improved accuracy over the individual schemes. Measured by precision, the superior model was TVFEMD-LSTM4, where the error measurements for the three stations were: RMSE = 0.101 m/s, MAE = 0.077 m/s, MAPE = 3.553 %, R^2^ = 0.995 at Carbondale, RMSE = 0.056 m/s, MAE = 0.044 m/s, MAPE = 3.154 %, R^2^ = 0.997 at Champaign, and RMSE = 0.160 m/s, MAE = 0.121 m/s, MAPE = 4.164 %, R^2^ = 0.993 at DeKalb. Performance of the standalone GB, RF, and LSTM models was not improved by increasing the number of lags; however, the outcomes of the hybrid models showed that accuracy for all stations was increased when the models had higher-lagged (WS) inputs. The run-times of the models were also investigated, and taking consideration of the trade-off between improved error metrics and lower computational time, the TVFEMD-GB-LSTM hybrid framework can generally be suggested as a promising tool.

## CRediT authorship contribution statement

**Shahab S. Band:** Project administration, Methodology. **Rasoul Ameri:** Formal analysis, Data curation. **Sultan Noman Qasem:** Supervision, Software. **Saeid Mehdizadeh:** Writing – review & editing, Formal analysis. **Brij B. Gupta:** Project administration, Methodology. **Hao-Ting Pai:** Writing – original draft, Visualization. **Danyal Shahmirzadi:** Writing – review & editing, Software. **Ely Salwana:** Data curation, Conceptualization. **Amir Mosavi:** Visualization, Validation.

## Data availability

The datasets used in this study are available from the corresponding author on reasonable request.

## Declaration of competing interest

The authors declare that they have no known competing financial interests or personal relationships that could have appeared to influence the work reported in this paper.
